# Physiological roles of regulated Ire1 dependent decay

**DOI:** 10.3389/fgene.2014.00076

**Published:** 2014-04-16

**Authors:** Dina S. Coelho, Pedro M. Domingos

**Affiliations:** Instituto de Tecnologia Química e Biológica, Universidade Nova de LisboaOeiras, Portugal

**Keywords:** Ire1, Xbp1, RIDD, endoplasmic reticulum stress, unfolded protein response

## Abstract

Inositol-requiring enzyme 1 (Ire1) is an important transducer of the unfolded protein response (UPR) that is activated by the accumulation of misfolded proteins in the endoplamic reticulum (ER stress). Activated Ire1 mediates the splicing of an intron from the mRNA of Xbp1, causing a frame-shift during translation and introducing a new carboxyl domain in the Xbp1 protein, which only then becomes a fully functional transcription factor. Studies using cell culture systems demonstrated that Ire1 also promotes the degradation of mRNAs encoding mostly ER-targeted proteins, to reduce the load of incoming ER “client” proteins during ER stress. This process was called RIDD (regulated Ire1-dependent decay), but its physiological significance remained poorly characterized beyond cell culture systems. Here we review several recent studies that have highlighted the physiological roles of RIDD in specific biological paradigms, such as photoreceptor differentiation in *Drosophila* or mammalian liver and endocrine pancreas function. These studies demonstrate the importance of RIDD in tissues undergoing intense secretory function and highlight the physiologic role of RIDD during UPR activation in cells and organisms.

## ENDOPLASMIC RETICULUM STRESS AND THE UNFOLDED PROTEIN RESPONSE

The endoplasmic reticulum (ER) is the entry site for the secretory pathway; all proteins targeted to the plasma membrane, extracellular space, and some organelles are translated into the ER, where they are folded and modified (Cooper, 2000). Proteins that fail to fold into their native conformation are targeted for ER-associated degradation (ERAD). Proteins marked as terminally misfolded are dislocated to the cytoplasm, where they are degraded by the ubiquitin-proteasome system (Smith et al., 2011; Claessen et al., 2012).

A number of cellular stress conditions such as low glucose levels, redox stress, or abnormal ER calcium content may perturb protein maturation in the ER or interfere with the capacity of the folding machinery in the ER (Marciniak and Ron, 2006). Many physiological processes may further challenge the ER by imposing suddenly large amounts of “client” proteins (Moore and Hollien, 2012). The imbalance between the ER folding capacity and the burden of incoming proteins may lead to the accumulation of misfolded proteins, causing ER stress. Adaptation to ER stress is mediated by the unfolded protein response (UPR; Ron and Walter, 2007; Hetz, 2012; Gardner et al., 2013). The UPR is a collection of integrated signaling pathways activated by ER-localized transmembrane protein sensors, which have luminal domains that detect misfolded proteins in the ER and cytoplasmatic effector domains that transduce signaling to the transcriptional and/or translational apparatus.

The UPR was first described in budding yeast, where it is represented by a single linear pathway (Mori et al., 1992). In higher eukaryotes the UPR is more complex and is mediated by three ER transmembrane sensors: pancreatic ER kinase (PKR)-like ER kinase (PERK), activating transcription factor 6 (ATF6), and inositol-requiring enzyme 1 (Ire1; Harding et al., 2002). The UPR outcomes are temporally coordinated: first, translation is attenuated to reduce the load of proteins into the ER; second, genes encoding ER chaperones and enzymes are up-regulated to increase the ER folding capacity; and third, genes encoding ERAD components are induced to enhance degradation of misfolded proteins from the ER.

If the mechanisms activated by the UPR are insufficient to decrease ER stress and restore ER homeostasis, cells may undergo apoptosis (Rasheva and Domingos, 2009). Chronic ER stress and defective activation of the UPR have been involved in the pathology of several human diseases, such as cancer, diabetes, neurodegenerative disorders, and chronic inflammation (Wang and Kaufman, 2012). Therefore, there has been increasing interest in controlling the ER stress pathways and discover new therapeutic targets to treat these diseases.

## Ire1 SIGNALING

**FIGURE 1:**
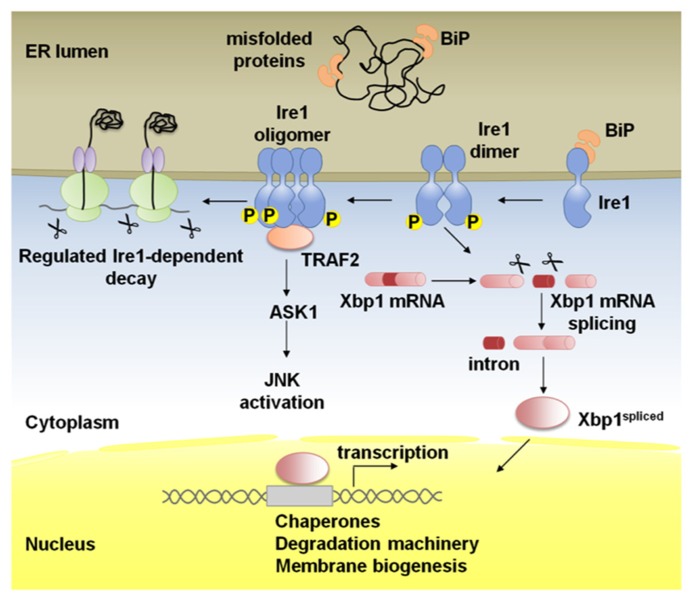
**Schematic representation of Ire1 signaling**. Binding immunoglobulin protein (BiP) binds Ire1 luminal domain and maintains it in a monomeric inactive form. In stressed cells, BiP is recruited to misfolded proteins and Ire1 is activated following conformational changes induced by dimerization of monomers in the plane of the membrane and trans-autophosphorylation. Higher order oligomers, which might form upon additional stress stimuli, reinforce Ire1 RNase activity. Activated Ire1 mediates the splicing of Xbp1 mRNA in higher eukaryotes (or Hac1 in yeast). Splicing of the intron from the Xbp1 transcript results in a frame-shift and the production of a potent transcription factor, Xbp1-spliced, that regulates many UPR target genes to promote protein folding in the ER lumen, ER-associated degradation (ERAD) of misfolded proteins and ER biogenesis. Ire1 can also act by alternative pathways; phosphorylated Ire1 associates with TRAF2 and activates the JNK pathway via ASK1. In the regulated Ire1 dependent decay (RIDD) pathway, Ire1 degrades mRNAs localized to the ER membrane through its RNase activity leading to a reduction in the amount of proteins imported into the ER lumen.

Being the most evolutionarily conserved arm of the UPR, Ire1 is a type I ER-resident transmembrane protein with a ER luminal dimerization domain and a cytoplasmic domain with Ser/Thr kinase and endoribonuclease activities (**Figure 1**; Cox et al., 1993; Mori et al., 1993; Shamu and Walter, 1996; Tirasophon et al., 1998; Wang et al., 1998; Liu et al., 2000; Koizumi et al., 2001; Shen et al., 2001; Korennykh and Walter, 2012).

In the budding yeast the only known substrate of Ire1 is the mRNA of the bZIP transcription factor Hac1 (Cox and Walter, 1996; Mori et al., 1996; Nikawa et al., 1996). In case of ER stress, Ire1 associates in oligomers after Binding immunoglobulin protein (BiP) release and activates its RNase domain by autophosphorylation (Shamu and Walter, 1996; Welihinda and Kaufman, 1996; Liu et al., 2000; Papa et al., 2003; Lee et al., 2008; Korennykh et al., 2009, 2011a). Activated Ire1 recognizes a double stem loop flanking a 252bp intron in Hac1 mRNA and cleaves it twice (Sidrauski and Walter, 1997; Korennykh et al., 2011b), while a transfer RNA ligase joins the exons (Sidrauski et al., 1996). This Ire1-mediated unconventional splicing event releases the translational repression exerted by the 252bp intron and allows the Hac1^spliced^ protein to act as a transcription factor (Chapman and Walter, 1997; Rüegsegger et al., 2001). The functional homolog of Hac1 in mammals is Xbp1 (Yoshida et al., 2001; Calfon et al., 2002), which is also only active as a transcription factor after the Ire1-mediated splicing of the Xbp1 mRNA. In this case, however, Xbp1^unspliced^ is translated and originates a protein that is rapidly degraded (Calfon et al., 2002; Yoshida et al., 2006).

Genetic profiling and analyses revealed that Hac1/Xbp1 control the expression of genes related to the UPR including chaperone induction, up-regulation of ERAD machinery, membrane biogenesis, and ER quality control (Lee et al., 2003; Shaffer et al., 2004; Shoulders et al., 2013). In mammals, Xbp1 also activates the expression of cell type specific targets linked to cell differentiation, signaling, and DNA damage (Acosta-Alvear et al., 2007; Lee et al., 2003).

## TARGETING OF mRNAs TO Ire1

The mechanism of recruitment of Hac1/Xbp1 mRNAs to the ER membrane seems to differ considerably between yeast and mammals. Under non-stressed conditions, unspliced Hac1 mRNA is found mostly in the cytoplasm, in association with stalled ribosomes. Upon ER stress, Hac1 mRNA is recruited to Ire1 clusters in the ER membrane, in a process that depends on translational repression and on a bipartite element (BE) present at the 3′ untranslated region of the Hac1 mRNA (Aragón et al., 2009).

In mammals, the Xbp1^unspliced^ mRNA is translated under normal conditions and originates a protein that associates with membranes via two hydrophobic regions (HR1 and HR2). The HR2 is a conserved region predicted to form a α-helix that has propensity to interact with the lipid bi-layer of the membrane (Yanagitani et al., 2009, 2011). Presumably, upon Xbp1 mRNA translation, HR1 and HR2 on the nascent polypeptide associate with the ER membrane and bring the Xbp1 mRNA-ribosome-nascent chain (RNC) complex to the vicinity of Ire1, facilitating Ire1-mediated splicing of Xbp1 mRNA.

## Xbp1 INDEPENDENT FUNCTIONS OF Ire1

Non-overlapping defects in Ire1 or Xbp1 mutant *Caenorhabditis elegans* first supported the existence of alternative roles for Ire1, besides Xbp1 mRNA splicing (Shen et al., 2005). Ire1 is thought to regulate apoptosis, autophagy, and ERAD through interaction with cytoplasmic partners independently of its RNase activity (Hetz and Glimcher, 2009). The cytosolic domain of Ire1 interacts with Traf2 (TNFR-associated factor 2) and activates ASK1 (Apoptosis signal-regulating kinase 1), triggering the JNK (cJun-N terminal kinase) pathway (Urano et al., 2000; Nishitoh et al., 2002). This Ire1/Traf2 interaction may lead to the activation of apoptosis under irreversible ER stress (Mauro et al., 2006). Ire1 may also control levels of autophagy under ER stress through activation of the JNK pathway (Ogata et al., 2006). The phosphorylation of the anti-apoptotic BCL2 at the ER by JNK stimulates autophagy by modulating the activity of Beclin1. Dissociation of the complex formed by BCL2 and Beclin1 induces autophagy (Pattingre et al., 2009).

The cytoplasmic domain of unphosphorylated (inactive) Ire1 physically interacts with the ubiquitin specific protease 14 (USP14), and this association is inhibited by ER stress and Ire1 activation (Nagai et al., 2009). USP14, which is recruited to the ERAD machinery via interaction with Ire1α, inhibits ERAD through a deubiquitination-independent mechanism (Nagai et al., 2009). Finally, non-muscle myosin IIB interacts with Ire1 during ER stress, revealing that interaction of Ire1 with the cytoskeleton is required for optimal regulation of Ire1 activity (He et al., 2012).

## REGULATED Ire1-DEPENDENT DECAY (RIDD)

A breakthrough report uncovered that the Ire1 RNase has broad range of mRNA substrates besides Xbp1 mRNA in *Drosophila* S2 cells. The group of Jonathan Weissman found through gene profiling experiments that a subset of mRNAs are degraded during ER stress by a mechanism that is dependent on Ire1 but not Xbp1 (Hollien and Weissman, 2006). The degraded mRNAs encoded mostly proteins with signal peptide/transmembrane domains that would represent an additional challenge to the ER folding machinery under ER stress. This mechanism was named RIDD, for Regulated Ire1-Dependent Decay, and was later also described in mammalian cells and in the fission yeast (which lacks any Hac1/Xbp1 homolog; Hollien et al., 2009; Cross et al., 2012; Kimmig et al., 2012). While in *Drosophila* S2 cells RIDD down-regulates many RNAs by 5-10 fold, in mammals the magnitudes of the changes in expression were smaller, twofold or less for many targets (Hollien and Weissman, 2006; Hollien et al., 2009).

The mechanism of targeting mRNAs to RIDD seems to have diverged throughout evolution (Hollien, 2013). In *Drosophila* S2 cells, RIDD has a broad scope of targets and there is a strong correlation between interaction of a mRNA with the ER membrane and the extension of its degradation by RIDD (Hollien and Weissman, 2006; Gaddam et al., 2013). In fact, deletion of the signal peptide from a known RIDD target prevents its degradation and conversely, addition of a signal peptide to GFP is sufficient to promote its degradation by RIDD. One interesting exception is the mRNA of PlexinA, which is strongly associated with the ER membrane, but it is protected from RIDD and is continuously translated, even during ER stress (Gaddam et al., 2013). PlexinA mRNA has regulatory upstream ORFs, which are necessary for its protection from RIDD. Another interesting exception is the mRNA encoding Smt3, a homolog of SUMO (small ubiquitin-like modifier), which is cleaved by RIDD on a stem loop structure, despite not being stably associated to the ER membrane (Moore et al., 2013).

In mammalian cells, RIDD targets are enriched for mRNAs containing a cleavage site with a consensus sequence (CTGCAG) and a predicted secondary structure similar to the conserved Ire1 recognition stem-loop of the Xbp1 mRNA (Han et al., 2009;Oikawa et al., 2010; Hur et al., 2012). Deletion of the stem-loop or mutagenesis of the conserved bases abrogated RIDD (Oikawa et al., 2010).

## PHYSIOLOGICAL RELEVANCE OF RIDD

The physiological relevance of RIDD has been recently demonstrated in several different biological models, with specific cellular and/or developmental conditions. RIDD has a role controlling the expression of lipogenic enzymes and modulating levels of lipids in the serum. Ire1β, which is specifically expressed in the epithelial cells of the gastrointestinal tract, has a protective role against hyperlipidemia in mice fed with a high fat or high cholesterol diet by decreasing the absorption of lipids in the intestine (Iqbal et al., 2008). Ire1β promotes the post-transcriptional degradation of the ER chaperone microsomal triglyceride transfer protein (MTP), involved in the assembly of apolipoproteins B and biosynthesis of chylomicrons (Iqbal et al., 2008). In the liver, Xbp1 deficiency provokes Ire1α hyperactivation, which contributes to a hypolipogenic phenotype in mice characterized by reduced plasma cholesterol and triglycerides (So et al., 2012). A comprehensive comparative microarray analysis identified 112 genes induced by Ire1α siRNA treatment in Xbp1-deficient mice. Among these genes are the ones encoding Angiopoietin-like protein 3 (Angptl3) and ces1 genes, which are involved in lipid metabolism and were further validated as RIDD substrates (So et al., 2012). The targeting of Xbp1 may be a therapeutic approach in dyslipidemic diseases, as Xbp1 deficiency in the liver, in leptin-deficient ob/ob mice, lowers hepatic triglycerides and plasma cholesterol levels (So et al., 2012).

Ire1β was also found to have a homeostatic role in the secretory goblet cells of the intestine through the down-regulation of mRNA levels of the major secretory product mucin 2 (Tsuru et al., 2013). The knock-out of Ire1β isoform in mouse colon results in disorganization of the ER in the goblet cells at early stages of maturation with accumulation of a precursor form of mucin 2 in the expanded ER lumen and induction of ER stress. Remarkably, Ire1α seems to have a distinct role in goblet cells mediating Xbp1 splicing and promoting the activation of UPR targets like BiP (Tsuru et al., 2013). Ire1α has a protective role in rodents against the liver damage caused by an overdose of the analgesic drug acetaminophen through the degradation of the mRNA of two P450 enzymes, Cyp1a2 and Cyp2e1, that are responsible for metabolizing the drug into a cytotoxic metabolite (Hur et al., 2012). Again, hyperactivation of Ire1α, caused by the liver specific deletion of Xbp1, prevents prolonged JNK activation and improves the morphology of the liver in mice injected with acetaminophen (Hur et al., 2012).

Several studies demonstrate that Ire1α plays an important role regulating pancreatic β-cells homeostasis by controlling the levels of insulin synthesized in the ER. Treatment of β cells with high levels of glucose hyperactivates Ire1, which correlates with a decrease of insulin mRNA expression (Lipson et al., 2008). Later, Ire1 was shown to cleave Insulin 1 and Insulin 2 mRNAs at specific sites *in vitro* (Han et al., 2009). Chronic stimulation of β-cells with high glucose concentrations might impose insurmountable levels of ER stress and promote the shift from a protective response (Xbp1 splicing and up-regulation of chaperones) to a deleterious response (RIDD and degradation of insulin). Supporting this hypothesis, islets from mice heterozygous for Ire1α are more resistant to chronic high glucose and had higher gene expression for both Insulin 1 and Insulin 2 (Lipson et al., 2008). Deletion of Ire1 may be beneficial in the case of diabetes type II models.

Maturation of insulin is also inefficient in β-cells deficient for Xbp1 due in part to RIDD. Ire1α is activated by Xbp1 silencing in Min6 insulinoma cells and activated Ire1α reduces the levels of components of the insulin secretory pathway, namely PC1, PC2, and CPE enzymes, by cleaving the respective mRNAs (Lee et al., 2011). Xbp1 deficient islets of the pancreas present morphological abnormalities, including disorganized structure, few insulin granules, and distended ER, consistent with Xbp1 being required for expression of ER chaperone genes such as BiP (Hspa5), ERdj4 (Dnajb9), and p58IPK (Dnajc3; Lee et al., 2011).

Regulated Ire1-dependent decay was also associated with innate and adaptive immunity. Ire1 is activated by binding part of the cholera toxin to induce an inflammatory response (Cho et al., 2013). In this case, Xbp1 is dispensable for signaling but RIDD is required for the activation of RIG-1 (retinoic acid inducible gene 1), NF-kB and interferon pathways (Cho et al., 2013). RIDD is necessary in CD8α^+^ dendritic cells for cross-presentation of cell-derived antigens via MHC-class I to CD8+ T cells (Osorio et al., 2014). Xbp1 is necessary to maintain a normal morphology of the ER in CD8α^+^ conventional dendritic cells, whereas RIDD has a critical function in regulating the expression of integrins and components of the major histocompatibility complex class I antigen-presentation machinery in these cells (Osorio et al., 2014). Moreover, RIDD is also active in B cells, where it cleaves the mRNA of secretory μ chains (Benhamron et al., 2014).

**FIGURE 2:**
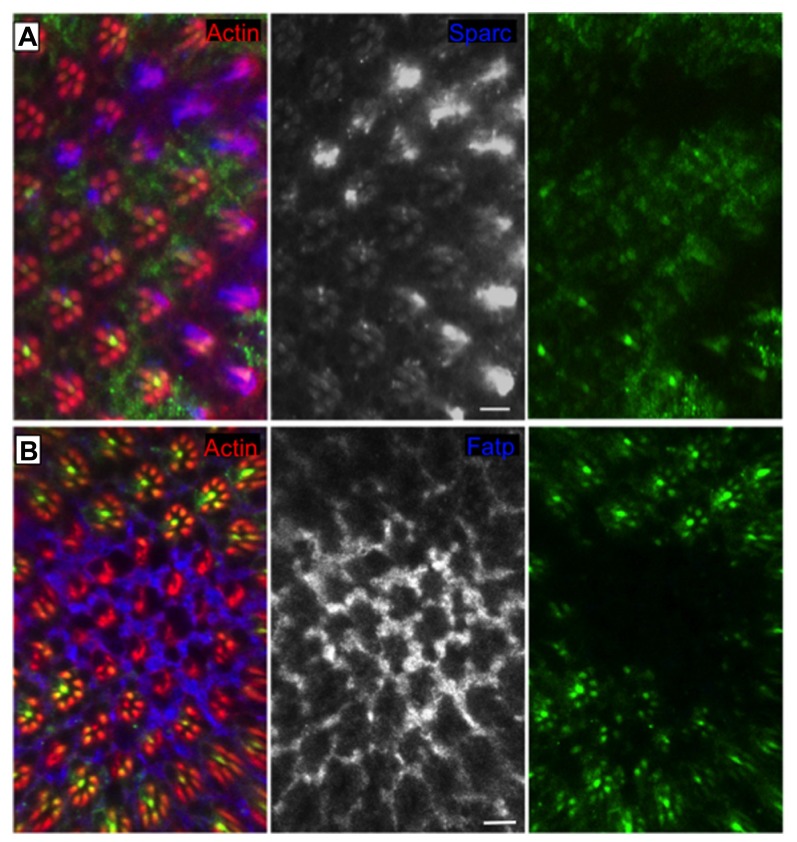
**RIDD “in action” in the *Drosophila* eye**. Clones of cells homozygous for Ire1 mutant chromosome (*PBac{WH}*Ire1^f02170^) are labeled by the absence of myrGFP. The protein levels of **(A)** Sparc (blue and monochrome) and **(B)** Fatp (blue and monochrome) are elevated in Ire1 mutant tissue, in comparison with the surrounding control tissue. Sparc and Fatp are two RIDD substrates in the *Drosophila* eye. Actin is in red. Scale bars represent 10 μm. Adapted from Coelho et al. (2013).

Regulated Ire1-dependent decay has an essential role in the differentiation of the photoreceptor cells in *Drosophila* to maintain the homeostasis of the secretory pathway during the morphogenesis of the light gathering organelle, the rhabdomere, which depends on the massive synthesis of membrane and proteins during the second half of pupal development (Coelho et al., 2013). Ire1 mutant photoreceptors show a very dramatic phenotype with atrophy of the rhabdomeres and collapse of the interrhabdomeral space (caused by a defect in the delivery of Rhodopsin1 and spacemaker protein, respectively), resulting in progressive degeneration of the retina in adult flies (Coelho et al., 2013). Remarkably, Xbp1 mutant photoreceptors show rhabdomeres with almost a normal morphology, evidencing Xbp1-independent roles of Ire1 in this specific biological context (Coelho et al., 2013). RIDD is activated in the *Drosophila* photoreceptors and promotes the degradation of several mRNA substrates (**Figure 2**), among them Fatp (Fatty acid transport protein), a previously described regulator of Rhodopsin1 levels in photoreceptor cells (Dourlen et al., 2012). Fatp mediates the uptake of fatty acids into cells and fatty acids are precursors for the biosynthesis of phosphatic acids. Increased levels of phosphatic acids were previously shown to disrupt rhabdomere morphogenesis (Raghu et al., 2009), causing a phenotype very similar to the one of Ire1 mutant photoreceptors.

Under conditions of overwhelming ER stress induction or chronic low level stress mRNAs encoding secretory cargo proteins and secretory pathway resident proteins start to decay (Han et al., 2009). Indeed, ER chaperone BiP and Golgi-localized glycosylating enzyme Gyltl1b are targets of RIDD. Unmitigated ER stress may deplete important cell surface proteins or secretory pathway proteins by continuous decay and promote apoptosis. Expression of wild-type Ire1 in Xbp1^-^^/^^-^ MEF triggers apoptosis, but expression of a Ire1 kinase active/RNase dead mutant does not induce apoptosis, arguing that an active RNase is required to induce pro-apoptotic signals independent of Xbp1 mRNA splicing (Han et al., 2009). Indeed, RIDD can also promote the cleavage of selected microRNAs (miRs – 17, 34a, 96, 125b) that normally repress translation of Caspase 2, to control induction of apoptosis upon continued ER stress (Upton et al., 2012).

The two Ire1 functions, RIDD and splicing of Xbp1 mRNA, can be uncoupled *in vitro*, allowing a better understanding of the physiological output of each pathway. The Ire1^I642G^mutant has an enlarged kinase pocket that prevents autophosphorylation and activation of the RNase. The need for ATP binding can be bypassed by incubation with 1NM-PP1, an ATP analog that binds specifically to the designed pocket of Ire1^I642G^ and activates the RNase by an allosteric mechanism (Papa et al., 2003; Hollien et al., 2009). Addition of the 1NM-PP1 is sufficient to induce Xbp1 splicing when Ire1^I642G^ is over-expressed, even in the absence of ER stress. However, RIDD function can only be engaged by 1NM-PP1 in the presence of ER stress, suggesting different activation modes of Ire1 (Han et al., 2009; Hollien et al., 2009). Other compounds, known as KIRAs (kinase inhibiting RNase attenuators) can bypass the need for autophosphorylation to activate wild-type Ire1, stimulating Xbp1 splicing, and tempering RIDD (Han et al., 2009).

## CONCLUSION

A variety of recent articles demonstrated that RIDD has several physiological roles in different experimental conditions and paradigms. In most cases, RIDD couples the load of ER targeted mRNAs with the ER folding capacity, maintaining the ER homeostasis during cell differentiation and ER expansion. In cases of extreme ER stress, RIDD may trigger apoptosis since it can promote the degradation of ER resident proteins and de-repress Caspase 2. As always, questions remain and should be addressed by additional studies. For example, is it possible to control the RIDD vs. Xbp1 splicing activities of Ire1 or to control the substrate specificity of RIDD to cause different biological outcomes? Stay tuned for further developments.

## Conflict of Interest Statement

The authors declare that the research was conducted in the absence of any commercial or financial relationships that could be construed as a potential conflict of interest.
